# Results from a large cross-sectional study assessing *Chlamydia trachomatis*, *Ureaplasma* spp. and *Mycoplasma hominis* urogenital infections in patients with primary infertility

**DOI:** 10.1038/s41598-021-93318-1

**Published:** 2021-07-01

**Authors:** Daniela Andrea Paira, Guillermo Molina, Andrea Daniela Tissera, Carolina Olivera, Rosa Isabel Molina, Ruben Dario Motrich

**Affiliations:** 1 Centro de Investigaciones en Bioquímica Clínica e Inmunología (CIBICI-CONICET), Facultad de Ciencias Químicas, Universidad Nacional de Córdoba, Haya de La Torre y Medina Allende, Ciudad Universitaria, X5016HUA Córdoba, Argentina; 2 Servicio de Urología y Andrología, Hospital Privado Universitario de Córdoba, 5016 Córdoba, Argentina; 3 Laboratorio de Andrología y Reproducción (LAR), 5000 Córdoba, Argentina

**Keywords:** Urogenital diseases, Bacterial infection, Infertility, Clinical microbiology

## Abstract

Female and male infertility have been associated to *Chlamydia trachomatis*, *Ureaplasma* spp. and *Mycoplasma hominis* urogenital infections. However, evidence from large studies assessing their prevalence and putative associations in patients with infertility is still scarce. The study design was a cross-sectional study including 5464 patients with a recent diagnosis of couple’s primary infertility and 404 healthy control individuals from Cordoba, Argentina. Overall, the prevalence of *C. trachomatis*, *Ureaplasma* spp. and *M. hominis* urogenital infection was significantly higher in patients than in control individuals (5.3%, 22.8% and 7.4% vs. 2.0%, 17.8% and 1.7%, respectively). *C. trachomatis* and *M. hominis* infections were significantly more prevalent in male patients whereas *Ureaplasma* spp. and *M. hominis* infections were more prevalent in female patients. Of clinical importance, *C. trachomatis* and *Ureaplasma* spp. infections were significantly higher in patients younger than 25 years. Moreover, *Ureaplasma* spp. and *M. hominis* infections were associated to each other in either female or male patients being reciprocal risk factors of their co-infection. Our data revealed that *C. trachomatis*, *Ureaplasma* spp. and *M. hominis* are prevalent uropathogens in patients with couple’s primary infertility. These results highlight the importance of including the screening of urogenital infections in the diagnostic workup of infertility.

## Introduction

Urogenital infections are known causes of infertility^1^. Currently, infertility affects 15–20% of reproductive-aged couples worldwide and women and men equally contribute to infertility cases^1,2^. Sexually transmitted infections can impair fertility by different mechanisms: by directly damaging organs and gametes and/or, indirectly, by the induced inflammation and associated tissue damage, scarring and obstruction^1,3^. Moreover, infection-induced genital inflammation may alter the normal immunomodulation process that naturally occurs in the female genital tract after mating to facilitate fertilization, embryo implantation and promote embryo growth for a successful pregnancy^4^. Besides being the most frequent sexually transmitted bacterial infection worldwide, *Chlamydia trachomatis* is a common infection associated to infertility^5^. In women, *C. trachomatis* is a known cause of different urogenital pathologies such as acute urethritis, cervicitis and salpingitis that may lead to severe reproductive complications including pelvic inflammatory disease, chronic pelvic pain, ectopic pregnancy, miscarriage and tubal infertility^6,7^. In men, *C. trachomatis* is considered the most common agent of non-gonococcal urethritis and may cause epididymitis-orchitis, prostatitis, sperm tract obstructions and alterations in sperm quality^8,9^. On the other hand, *Mycoplasma hominis,* and *Ureaplasma urealyticum* and *Ureaplasma parvum* (the latter being the only two *Ureaplasma* spp. associated to humans) have also been recognized as sexually transmitted infections that could impair human fertility^1,10^. Although they are known to colonize the female and male reproductive tracts as commensals, cumulative growing evidence has shown they are emerging sexually transmitted opportunistic pathogens able to cause asymptomatic chronic disorders affecting female and male fertility^10–16^. In men, *Ureaplasma* spp. and *M. hominis* are causes of non-gonococcal urethritis contaminating semen during ejaculation. Moreover, *Ureaplasma* spp. have been proposed to cause prostatitis, epididymitis and infertility^1,10^. In addition, reported data have shown that both *Ureaplasma* spp. and *M. hominis* could impair sperm quality^10,12,17,18^. In women, *Ureaplasma* spp. and *M. hominis* may cause different pathologies including acute urethritis, bacterial vaginosis, pelvic inflammatory disease and tubal infertility^15,19,20^. Moreover, the asymptomatic infection by mycoplasmas or ureaplasmas could induce pro-inflammatory immune responses in the endometrium that may impair pregnancy outcomes^2,9,15,21^.

The detection rates of *C. trachomatis*, *Ureaplasma* spp. and *M. hominis* in the urogenital tract form infertile women and men has shown striking variations across regions and countries and in different groups when individuals were classified according to age, ethnicity and socioeconomic status^5,22–24^. In that regard, a growing number of studies have been reported during the last decade. However, compelling available data from large cross-sectional studies is scarce^22–24^. Moreover, reported data about the association of these infections in either infertile women or men is limited^22–24^. Since these infections may play a significant role in the etiology of infertility, we herein conducted a large observational investigation into urogenital *C. trachomatis*, *Ureaplasma* spp. and *M. hominis* infections in women and men seeking care for couple’s primary infertility. Moreover, we analyzed the associations among infections and with demographic parameters such as patient sex and age.

## Results

### Prevalence of *C. trachomatis*, *Ureaplasma* spp. and *M. hominis* urogenital infection in patients with couple’s primary infertility

A total of 5164 patients (1554 women and 3610 men) with a recent diagnosis of couple’s primary infertility undergoing initial infertility evaluation and 404 control individuals (64 women and 340 men) were enrolled in the study. The overall prevalence of urogenital *C. trachomatis*, *Ureaplasma* spp. and *M. hominis* infection was significantly higher in patients than in control individuals (5.3%, 22.8% and 7.4% vs. 2.0%, 17.8% and 1.7%, respectively, Table 1). In females, *Ureaplasma* spp. and *M. hominis* infections were significantly more prevalent in patients than in controls (31.1% and 12.1% vs. 14.1% and 1.6%, respectively) whereas in males *C. trachomatis* and *M. hominis* infections were significantly more prevalent in patients than in controls (5.8% and 5.3% versus 1.8% and 1.8%, respectively) (Table 1).

**Table 1:** Prevalence of urogenital infections in patients with primary couple's infertility and control individuals.

	Total			Women			Men		
	Patients	Controls	*P*	Patients	Controls	*p*	Patients	Controls	*p*
*Chlamydia trachomatis (%)*	5.3	2.0	**0.0032**	4.3	3.1	0.6453	5.8	1.8	**0.0019**
*Ureaplasma* spp. *(%)*	22.8	17.8	**0.0215**	31.1	14.1	**0.0036**	19.2	18.5	0.7005
*Mycoplasma hominis (%)*	7.4	1.7	**< 0.0001**	12.1	1.6	**0.0087**	5.3	1.8	**0.0041**

Chi-square test. A *p < 0.05 was considered statistically significant.

Bold *p* numbers mean those which are statistically significant (<0.05).

### Demographic parameters associated to *C. trachomatis*, *Ureaplasma* spp. and *M. hominis* infection in patients with couple’s primary infertility

When analyzing the prevalence of infections within the patient population, it was found that *C. trachomatis*, *Ureaplasma* spp. and *M. hominis* infections were significantly associated with patient sex and age (Table 2). In fact, univariate regression analysis revealed that *C. trachomatis* infection was more likely to be detected in male than in female patients with an odds ratio of 1.36 (95% CI: 1.02–1.80, *p* = 0.034) (Table 2). Moreover, a significant association was particularly found between *C. trachomatis* infection and male patients younger than 25 years (OR: 2.51, 95% CI: 1.40–4.48, *p* = 0.002, Table 2) indicating that men, and especially those younger than 25 years, are at higher risk of infection than women (Table 2). Multivariate analysis further confirmed these associations (Supplementary Table S1).

**Table 2:** Prevalence of infections in patients with couple’s primary infertility according to sex and age.

Variables	Patients	*C. trachomatis* infection				*Ureaplasma* spp. infection				*M. hominis* infection			
	n	n (prevalence)	Odds ratio	95% CI	*P*	n (prevalence)	Odds ratio	95% CI	*p*	n (prevalence)	Odds ratio	95% CI	*p*
**Sex**													
Women	1554	67 (4.3%)	1.00 (ref.)			484 (31.1%)	1.00 (ref.)			188 (12.1%)	1.00 (ref.)		
Men	3610	208 (5.8%)	1.36	1.02–1.80	0.034 *	692 (19.2%)	0.52	0.46–0.60	< 0.001 *	192 (5.3%)	0.41	0.33–0.50	< 0.001*
**Age (y.o.)**													
*Women*													
> 40	355	17 (4.8%)		1.00 (ref.)		107 (30.1%)	1.00 (ref.)			43 (12.1%)	1.00 (ref.)		
40–25	1130	47 (4.2%)	0.94	0.50–1.78	0.847	341 (30.2%)	0.88	0.66–1.17	0.372	135 (11.9%)	0.80	0.55–1.19	0.275
< 25	69	3 (4.3%)	0.96	0.26–3.50	0.949	36 (52.2%)	2.27	1.33–3.89	0.003*	10 (14.5%)	1.04	0.49–2.22	0.910
*Men*													
> 40	1356	88 (6.5%)		1.00 (ref.)		233 (17.2%)	1.00 (ref.)			72 (5.3%)	1.00 (ref.)		
40—25	2161	106 (4.9%)	0.71	0.52–0.95	0.021*	433 (20.0%)	1.26	1.04–1.51	0.016*	16 (5.4%)	1.03	0.75–1.41	0.863
< 25	93	14 (15.1%)	2.51	1.40–4.48	0.002*	26 (28.0%)	1.66	1.04–2.66	0.034*	4 (4.3%)	0.72	0.26–2.02	0.532

Univariate analysis. 95%CI: 95% confident interval. A *p < 0.05 was considered statistically significant.

Bold *p* numbers mean those which are statistically significant (<0.05).

On the contrary, *Ureaplasma* spp. and *M. hominis* infections were associated to female patients, since male patients were less likely at risk of *Ureaplasma* spp. and *M. hominis* infection than females with odds ratios of 0.52 (95% CI: 0.46–0.60, *p* < 0.001, Table 2) and 0.41 (95% CI: 0.33–0.50, *p* < 0.001, Table 2), respectively. In addition, it was found that *Ureaplasma* spp. was more prevalent in patients younger than 25 years, either in women or men (OR: 2.27, 95% CI: 1.33–3.89, *p* = 0.003, and OR: 1.66, 95% CI: 1.04–2.66, *p* = 0.034, respectively; Table 2). Multivariate analysis further confirmed these data (Supplementary Table S1).

These results indicate that *C. trachomatis*, *Ureaplasma* spp. and *M. hominis* urogenital infections are associated with patient sex and age. In fact, patients younger than 25 years at the highest risk of *C. trachomatis* and *Ureaplasma* spp. infection. Moreover, our data show that male patients are at higher risk of *C. trachomatis* infection and, conversely, female patients are at higher risk of *Ureaplasma* spp. and *M. hominis* infection.

### Associations among *C. trachomatis*, *Ureaplasma* spp. and *M. hominis* urogenital infections in patients with couple’s primary infertility

**Figure 1:**
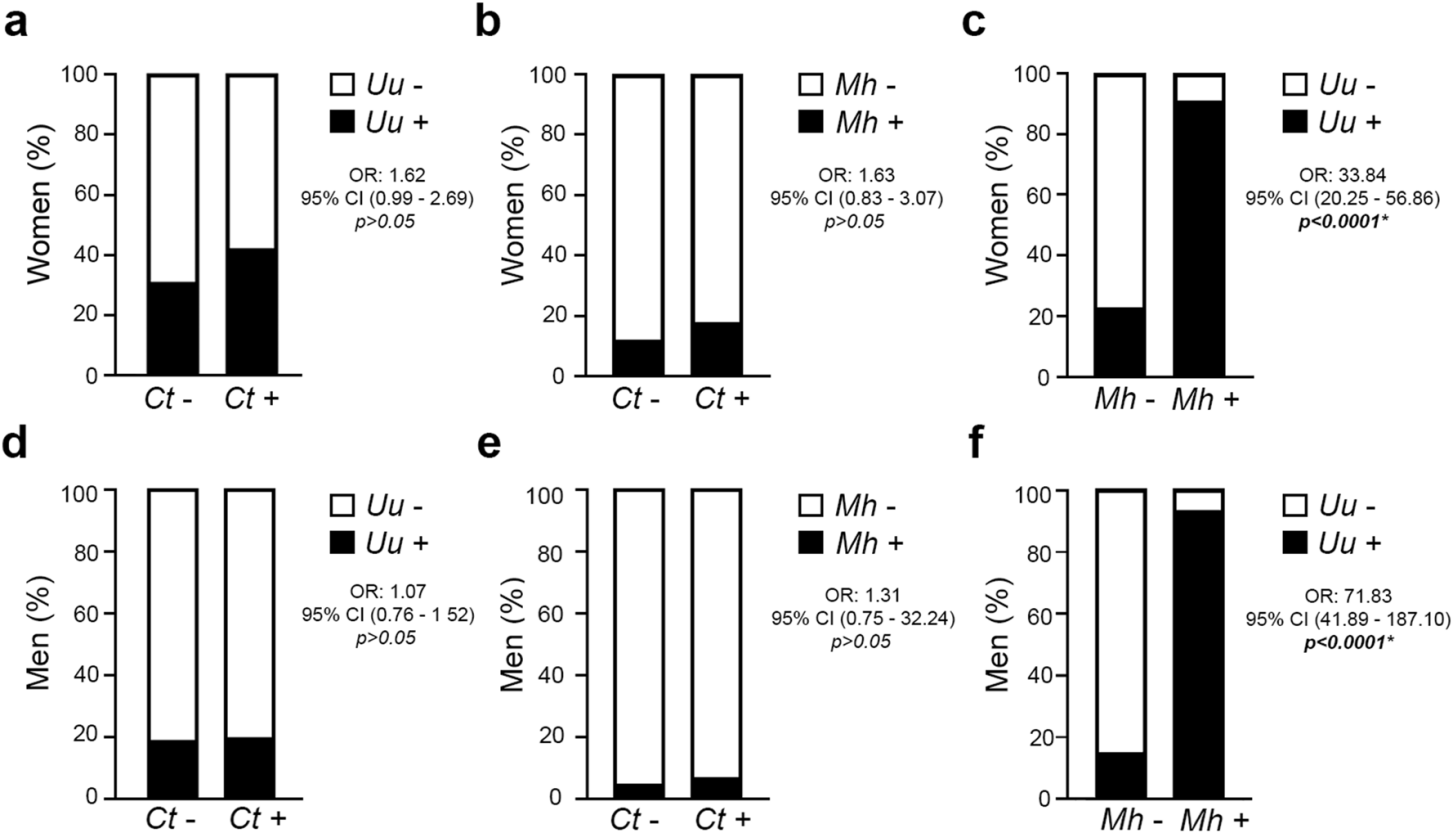
*C. trachomatis *(*Ct*)*, Ureaplasma* spp*. *(*Uu*)* and M. hominis *(*Mh*)* co-infections in patients with couple’s primary infertility.* Frequency of positive Uu infection in Ct-infected (Ct+) or Ct-non infected (Ct−) female (**a**) or male (**d**) patients. Frequency of positive Mh infection in Ct-infected (Ct+) or Ct-non infected (Ct−) female (**b**) or male (**e**) patients. Frequency of positive Uu infection in Mh-infected (Mh+) or Mh-non infected (Mh−) female (**c**) or male (**f**) patients. Data are shown as frequency. Chi square test were assessed and odds ratio with 95% confident interval (OR, 95% CI) calculated. A *p < 0.05 was considered statistically significant.

When assessing the co-infection between *C. trachomatis* and *Ureaplasma* spp. within the patient population, no significant association was found in either women (OR: 1.62, 95% CI: 0.99–2.69, *p *> 0.05) or men (OR: 1.07, 95% CI: 0.76–1.52, *p* > 0.05) (Fig. 1a, d). In fact, a similar prevalence of *Ureaplasma* spp. was found in *C. trachomatis* infected (41.8%, 28/67) and *C. trachomatis* non-infected (30.7%, 456/1487) female patients (Fig. 1a, Table 3). In addition, a comparable prevalence of *Ureaplasma* spp. were found in *C. trachomatis* infected (20.2%, 42/208) and *C. trachomatis* non-infected (19.1%, 650/3402) male patients (Fig. 1d, Table 3).

**Table 3:** *C. trachomatis*, *Ureaplasma* spp. and *M. hominis* co-infections in patients with couple’s primary infertility.

	*C. trachomatis*				*M. hominis*			
	Women		Men		Women		Men	
	Positive	Negative	Positive	Negative	Positive	Negative	Positive	Negative
	(n = 67)	(n = 1487)	(n = 208)	(n = 3402)	(n = 188)	(n = 1366)	(n = 192)	(n = 3418)
***Ureaplasma* spp.**								
Positive n (%)	28 (41.8%)	456 (30.7%)	42 (20.2%)	650 (19.1%)	171 (90.9%) *	313 (22.9%)	178 (92.7%) *	514 (15.0%)
Negative n (%)	39 (58.2%)	1031 (69.3%)	166 (79.8%)	2752 (80.9%)	17 (9.1%)	1053 (77.1%)	14 (7.3%)	2904 (85.0%)
***M. hominis***								
Positive n (%)	12 (17.9%)	176 (11.8%)	14 (6.7%)	178 (5.2%)	–	–	-	-
Negative n (%)	55 (82.1%)	1311 (88.2%)	194 (93.3%)	3224 (94.8%)	–	–	-	-

Univariate analysis. 95%CI: 95% confident interval. A *p < 0.05 was considered statistically significant.

Bold *p* numbers mean those which are statistically significant (< 0.05).

Likewise, there was no significant association between *C. trachomatis* and *M. hominis* infections in either female patients (OR: 1.63, 95% CI: 0.83–3.07, *p* > 0.05) or male patients (OR: 1.31, 95% CI: 0.75–32.24, *p* > 0.05) (Fig. 1b, e). From the 67 female patients infected with *C. trachomatis*, 12 were positive for *M. hominis* (17.9%), whereas 176 out of the 1487 *C. trachomatis*-negative female patients were positive for *M. hominis* (11.8%) (Fig. 1b, Table 3). Also, a comparable prevalence of *M. hominis* was found in either *C. trachomatis* infected (6.7%, 14/208) or *C. trachomatis* non-infected (5.2%, 178/3402) male patients (Fig. 1e, Table 3).

Interestingly, a significant association between *M. hominis* and *Ureaplasma* spp. infection was found in female patients (OR: 33.84, 95% CI: 20.25–56.86, *p* < 0.0001) as well as in male patients (OR: 71.83, 95% CI: 41.89–187.10, *p* < 0.0001) (Fig. 1c, Table 3). As detailed in Table 3, from the 188 female patients positive for *M. hominis*, 171 (91.0%) were positive for *Ureaplasma* spp. Similarly, a significantly increased prevalence of *Ureaplasma* spp. (92.7%) was found in *M. hominis* positive male patients (Fig. 1f). In fact, from the 192 male patients positive for *M. hominis* detection, 178 (92.7%) were positive for *Ureaplasma* spp. (Table 3). Multivariate regression analysis further confirmed these tight associations, indicating that *Ureaplasma* spp. and *M. hominis* act as mutual risk factors of infection in either female or male patients (Supplementary Table S1).

Noteworthy, only 2.0% of infected female patients (11 out of 539) and 1.5% of infected male patients (13 out of 871) were co-infected with the three uropathogens analyzed (Supplementary Figure S1).

## Discussion

*Chlamydia trachomatis*, *Ureaplasma* spp. and *M. hominis* are amongst the most frequent sexually transmitted bacterial infections. Moreover, they have been associated to infertility in either females or males^1–3,10,11,23^. Therefore, compelling data about the prevalence of urogenital *C. trachomatis*, *Ureaplasma* spp. and *M. hominis* infection and their possible associations in partners of infertile couples is of utmost importance. It has been shown that the prevalence of urogenital *C. trachomatis* infection in infertile men and women varies considerable across nations and regions and according to the subject population under study^25,26^. Besides, although a growing number of studies about the prevalence of urogenital *Ureaplasma* spp. and *M. hominis* infections in infertile patients have been reported during the last decade, compelling available data from large cross-sectional studies is still scarce^22–24^. Thus, we herein conducted a large observational investigation into urogenital *C. trachomatis*, *Ureaplasma* spp. and *M. hominis* infections in women and men with couple’s primary infertility undergoing initial infertility evaluation. Our data revealed a significantly higher overall prevalence of *C. trachomatis*, *Ureaplasma* spp. and *M. hominis* infection in patients with respect to control individuals, being *C. trachomatis* and *M. hominis* significantly more prevalent in male patients than in control men and *Ureaplasma* spp. and *M. hominis* significantly more prevalent in female patients than in control women. When analyzing the prevalence of infections within the patient population, univariate and multivariate regression analyses revealed that *C. trachomatis* infection was significantly more prevalent in males whereas *Ureaplasma* spp. and *M. hominis* infections were significantly more prevalent in females. The higher prevalence of *C. trachomatis* urogenital infection we found in male patients with respect to females could be due to the fact that urogenital infections in men are much more frequently asymptomatic than in women^8^. Besides, our results are in line with recently reported data showing higher prevalence of *M. hominis* and *Ureaplasma* spp. urogenital infections in infertile patients, especially in women^18,27–29^. Furthermore, our results also showed that *C. trachomatis* and *Ureaplasma* spp. infections were significantly more prevalent in young patients, particularly in those younger than 25, indicating that age as a risk factor and in agreement with previously reported data^30,31^. In fact, it is known that sexually transmitted infections are directly related to sexual experience, having young people more frequent sexual intercourses, less consistency of condom use and one or multiple sexual partners^30,31^. On the other hand, when assessing co-infections, only 2.0% of female patients and 1.5% of male patients were co-infected with the three uropathogens analyzed.

Our results support previously reported data. In a large observational study, Chen et al*.* found a prevalence of *C. trachomatis* infection of 3.5% in a population of 666 women seeking care for assisted reproduction in China, being significantly highest in patients younger than 25 years^32^. Moreover, Piscopo et al*.* reported a prevalence of *C. trachomatis* infection of 3.7% in women with tubal infertility in Brazil^28^. Besides and similar to our data, a prevalence of *C. trachomatis* infection of 4.3% in infertile men from Jordan has been reported^33^. Moreover, our results are in line with reported data by Sleha et al*.*, who found a prevalence of *U. urealyticum* and *M. hominis* urogenital infection of 39.6% and 8.1%, respectively, in Czech women undergoing an initial infertility evaluation^34^. Interestingly, a recent meta-analysis showed a significant association between *M. hominis* and *U. urealyticum* infections and female infertility^29^. In addition, Gdoura et al*.* found a prevalence of *U. urealyticum* and *M. hominis* of 15.4% and 9.6% in male partners from infertile couples from Tunisia^35^. Similar data were also reported in infertile men from China^36^.

However, significantly different prevalence rates of urogenital *C. trachomatis*, *Ureaplasma* spp. or *M. hominis* infection in infertile women or men have been reported in other studies. In comparison with our data, a much lower prevalence of *Ureaplasma* spp. infection was described in male partners of infertile couples from Italy^37^. Similarly, Boeri et al*.* recently reported lower prevalences of *C. trachomatis* and *M. hominis* infection in Italian men with primary infertility^38^. Moreover, in a study conducted in Spain, Veiga et al*.* found lower rates of *C. trachomatis*, *Ureaplasma* spp. and *M. hominis* infection in male partners of infertile couples than ours (0.9%, 15.1% and 0.9%, respectively)^39^. In addition, considerably lower prevalence rates of *C. trachomatis*, *Ureaplasma* spp. and *M. hominis* infection were reported in female partners of infertile couples from the USA^40^. Several factors could underlie these differences such as disparities in population under study (geographical location, age, ethnicity, religion, socio-economic status, access to medical care, etc.) or study design [prospective versus retrospective, patient population size, methods used for infection diagnosis (culture, PCR, etc., qualitative versus quantitative), etc.]. However, our results are in line with cumulative reported data and supported by the large patient population analyzed, including 5164 infertile individuals (1554 women and 3610 men).

Besides, our data revealed that *C. trachomatis* infection was not associated to either *Ureaplasma* spp. or *M. hominis* in female as well as in male patients. Conversely, and supporting recently reported data^41–43^, *Ureaplasma* spp. or *M. hominis* were significantly associated to each other in either female or male patients, indicating that *Ureaplasma* spp. or *M. hominis* urogenital infection increased the risk of *M. hominis* or *Ureaplasma* spp. co-infection. This tight infection association could be due to shared infection routes and/or pathophysiologic mechanisms^1,11^.

To our knowledge, this is the first cross-sectional study to investigate the prevalence and association of *C. trachomatis*, *Ureaplasma* spp. and *M. hominis* in patients with couple’s primary infertility from Argentina. Being one of the few cross-sectional study performed in Latin American countries^22,24^ and the large number of patients analyzed are the main strengths of our study. However, our study has some limitations such as the relatively smaller number of controls included, especially women. Moreover, we did not have the information of the partner couple of every female or male patient enrolled, which could have provided important information about infection concordance.

In conclusion, our results indicate that urogenital *C. trachomatis*, *Ureaplasma* spp. and *M. hominis* infections are prevalent in patients with couple’s primary infertility. *C. trachomatis* and *M. hominis* infections were significantly more prevalent in male patients whereas *Ureaplasma* spp. and *M. hominis* infections were more prevalent in female patients. Of clinical importance, *C. trachomatis* and *Ureaplasma* spp. infections were more prevalent in young patients, especially in those younger than 25 years. Moreover, *Ureaplasma* spp. and *M. hominis* showed to be reciprocal risk factors of their co-infection in either female or male patients. Overall, these results point out the importance to include the microbiological screening of urogenital infections in the diagnostic workup for infertility. Moreover, they highlight the need to reinforce preventive strategies at the primary healthcare level. Increasing awareness among people and health care practitioners are efficient approaches for the prevention of infection transmission. Future research is needed to unveil the true impacts of these uropathogens and the underlying pathophysiological mechanisms, which would allow the identification of proper and efficient treatments to more effectively reduce the burden of infertility.

## Methods

### Study design, patients and samples

This cross-sectional study was performed on a total cohort of 5164 consecutive Argentinian patients (1554 females and 3610 males) with a recent diagnosis of couple’s primary infertility undergoing initial infertility evaluation and in 404 healthy control individuals (64 women and 340 men) assessed at a joint Academic, Urology and Reproduction Health Center between January 2015 and November 2019. Expert, infertility-trained gynecologists or uro-andrologists comprehensively evaluated patients and primary infertility was diagnosed according to the World Health Organization (WHO) criteria, i.e. when a couple was unable to conceive a pregnancy after at least 12 months of unprotected intercourse^44^. Patients did not have any signs or symptoms of genital tract infections (urethral or vaginal discharge, dysuria, urethral irritation, itching, genital lesions, and pelvic/abdominal pain). Control individuals were seemingly healthy women or men attending the center during the same study period to receive regular annual physical, gynecological and/or urological check-up without fertility-related complaints and clinically asymptomatic for any infection. Inclusion criteria for either patients or controls were female or male aged 18–60 years, and not taking antibiotics when sampling and during the last 3 weeks. Cervicovaginal-swab and semen samples were collected from female and male individuals, respectively. Well-trained and experienced operators collected different cervicovaginal swabs for the detection of *C. trachomatis* or *Ureaplasma* spp. and *M. hominis*. Dacron swabs were inserted into the vagina up to the vault, rotated in the vaginal vault and vigorously scrubbed the mucous lining. The specimens were immediately transferred to tubes containing specific transport media and processed for analysis. Semen samples were collected by masturbation after 2–7 days of sexual abstinence and processed within 1 h of collection.

### Ethical approval

The study was carried out in accordance with The Code of Ethics of the World Medical Association (Declaration of Helsinki) standards and the Argentinian legislation for protection of personal data (Law 25326). The experimental protocol was approved by the Institutional Ethics Committee from the Hospital Nacional de Clinicas, Universidad Nacional de Cordoba (RePIS #3512). All patients and controls provided a signed written informed consent form agreeing to share their own anonymous information.

### DNA extraction and *C. trachomatis* detection

Total DNA was extracted from vaginal or semen samples and *C. trachomatis* infection detected by polymerase chain reaction (PCR) using the *C. trachomatis* 330/740 IC PCR kit (Sacace Biotechnologies Srl, Como, Italy) and following the manufacturer’s instructions. Analyses were performed within 4 h of sample collection.

### *Mycoplasma hominis* and *Ureaplasma* spp. infection assessment

*Mycoplasma hominis* or *Ureaplasma* spp. detection and enumeration were assessed by culture using the commercially available Complement Mycofast RevolutioN assay (ELITech MICROBIO, Signes, France) following the manufacturer’s instructions. Briefly, vaginal swabs or semen samples were inoculated in UMMt transport medium that contains preservatives and selective agents to inhibit the growth of contaminating flora. This medium was then dispensed into test wells, covered with two drops of mineral oil, sealed and incubated at 37 °C ± 1 °C for 24–48 h and observed for color changes. According to the kit specifications and as previously indicated^10,45–47^, a positive result was recorded when orange or red color changes were observed, indicating the presence of *M. hominis* and/or *Ureaplasma* spp., respectively, at a load of each microorganism ≥ 10^4^ CFU/ml.

### Statistics

Statistical analysis was performed using IBM SPSS statistical software, version 17.0 (SPSS Inc., Chicago, IL, USA) and GraphPad Prism 8.0.1 (GraphPad Inc., San Diego, CA, USA). Infection prevalences between patient and control groups were compared using Chi-Square test. Demographic characteristics (age, sex) and co-infections between infected and non-infected patients were compared using the chi-square test and the odds ratios (OR) and 95% confidence intervals (CI) were calculated. Univariate and multivariate logistic regression analyses for all variables were performed to determine associations or risk factors for infections. A *p* < 0.05 was considered statistically significant.

## Supplementary Information

Supplementary Information.
