# Supplementary material for: Results from a large cross-sectional study assessing Chlamydia trachomatis, Ureaplasma spp. and Mycoplasma hominis urogenital infections in patients with primary infertility
Source: Sci Rep. 2021 Jul 1;11:13655. doi: 10.1038/s41598-021-93318-1 (PMC8249471; doi:10.1038/s41598-021-93318-1)
Supplement: Supplementary file 1 — Supplementary Information. [file 41598_2021_93318_MOESM1_ESM.pdf]

Results from a large cross-sectional assessing *Chlamydia trachomatis*, *Ureaplasma* spp. and *Mycoplasma hominis* urogenital infections in patients with primary infertility.

Daniela A. Paira [1], Guillermo Molina [2], Andrea Tissera [3], Carolina Olivera [1], Rosa I. Molina [3], Ruben D. Motrich [1]*.

[1] Centro de Investigaciones en Bioquímica Clínica e Inmunología (CIBICI-CONICET), Facultad de Ciencias Químicas, Universidad Nacional de Córdoba, 5016, Córdoba, Argentina.

[2] Servicio de Urología y Andrología. Hospital Privado Universitario de Córdoba, 5016, Córdoba, Argentina.

[3] Laboratorio de Andrología y Reproducción (LAR), 5000, Córdoba, Argentina.

* Corresponding author: Ruben D. Motrich. CIBICI-CONICET. Facultad de Ciencias Químicas. Universidad Nacional de Córdoba. Haya de la Torre y Medina Allende. Ciudad Universitaria. Córdoba. X5016HUA. Argentina.

Phone: +54-351-535-3882 ext. 3152. E-mail: rmotrich@unc.edu.ar

https://orcid.org/0000-0002-5772-1285

Supplementary Table S1. Multivariate analysis of risk factors for *C. trachomatis*, *Ureaplasma* spp. or *M. hominis* urogenital infection in patients with couple's primary infertility

		***C. trachomatis* infection**					***Ureaplasma* spp. infection**					***M. hominis* infection**				
**Variables**	n	Positive	Negative	Odds ratio	95% CI	*p*	Positive	Negative	Odds ratio	95% CI	*p*	Positive	Negative	Odds ratio	95% CI	*p*
**Age (yo)**																
> 40	1404	89	1315	1.00 (referent)			276	1128	1.00 (referent)			97	1307	1.00 (referent)		
40 - 25	3587	167	3420	0.75	0.57-0.98	**0.036 ***	838	2749	1.20	1.01-1.43	**0.046 ***	269	3318	0.84	0.64-1.11	0.228
< 25	173	19	154	1.91	1.13-3.24	**0.016 ***	62	111	2.38	1.64-3.45	**<0.001 ***	14	159	0.51	0.27-0.96	**0.039 ***
**Sex**																
Women	1554	67	1487	1.00 (referent)			484	1070	1.00 (referent)			188	1366	1.00 (referent)		
Men	3610	208	3402	1.38	1.03-1.84	**0.030 ***	692	2918	0.64	0.54-0.74	**<0.001 ***	192	3418	0.57	0.44-0.71	**<0.001 ***
**Infection**																
C. trachomatis																
Negative	4889	-	-				1106	3783	1.00 (referent)			354	4535	1.00 (referent)		
Positive	275	-	-				70	205	1.10	0.80-1.53	0.548	26	249	1.27	0.78-2.06	0.340
Ureaplasma spp.																
Negative	3988	205	3783	1.00 (referent)			-	-				31	3957	1.00 (referent)		
Positive	1176	70	1106	1.10	0.79-1.53	0.560	-	-				349	827	51.53	35.70-75.08	**<0.001 ***
M. hominis																
Negative	4784	249	4535	1.00 (referent)			827	3957	1.00 (referent)			-	-			
Positive	380	26	354	1.33	0.82-2.16	0.247	349	31	51.57	35.39-75.13	**<0.001 ***	-	-			

95%CI: 95% confident interval. A *p<0.05 was considered statistically significant.

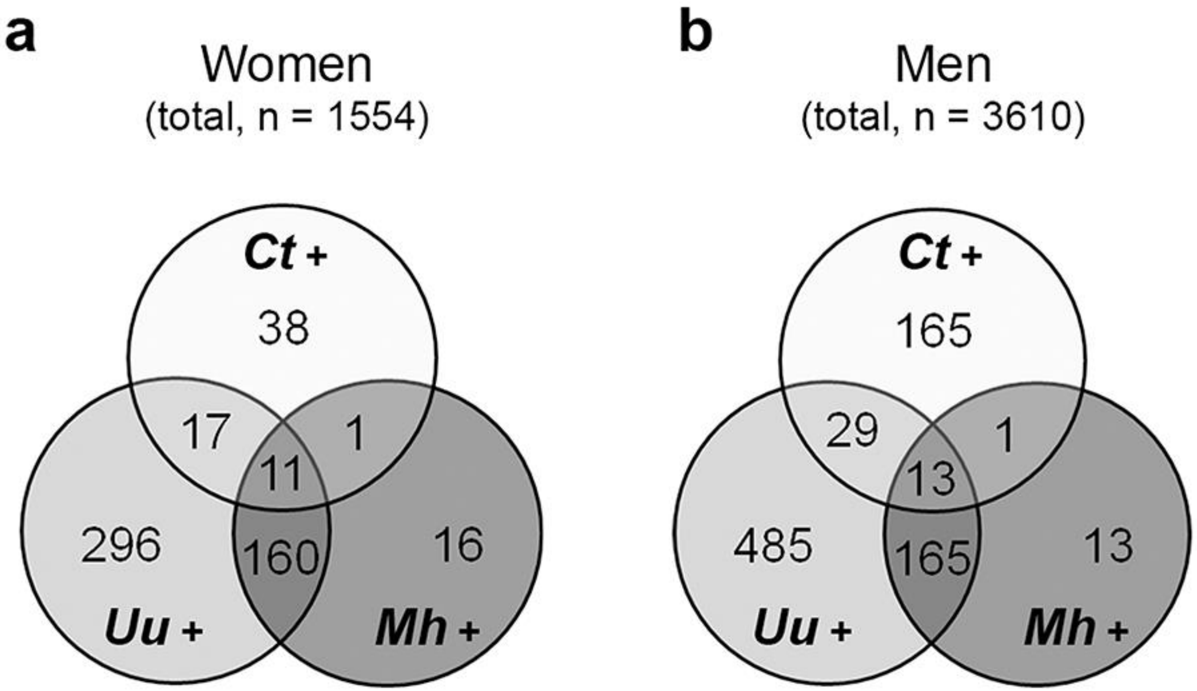

Supplementary Figure S1: *Representative scheme of C. trachomatis, Ureaplasma spp. and M. hominis infections and co-infections in patients with couple's primary infertility.* Venn diagrams represents three main groups that correspond to the uropathogens analyzed: *C. trachomatis* (Ct), *Ureaplasma* spp. (Uu) and *M. hominis* (Mh). The unions of the sets symbolize the presence of the respective co-infections. Data are shown as number of female (**a**) and male (**b**) patients.
